# MRI-based synthetic CT for assessment of the bony elements of the sacroiliac joints in children

**DOI:** 10.1186/s13244-023-01603-6

**Published:** 2024-02-18

**Authors:** Eva Schiettecatte, Elke Vereecke, Jacob L. Jaremko, Lieve Morbée, Caroline Vande Walle, Lennart Jans, Nele Herregods

**Affiliations:** 1 Department of Radiology and Nuclear Medicine, Ghent University Hospital, Corneel Heymanslaan 10, 9000 Ghent, Belgium; 2 Department of Radiology, University of Alberta Hospital, 8440-112 Street, Edmonton, Alberta T6G 2B7 Canada

**Keywords:** Sacroiliac joints, Pediatrics, Computed tomography, Magnetic resonance imaging, Artificial intelligence

## Abstract

**Objectives:**

The purpose of this study is to assess the equivalency of MRI-based synthetic CT (sCT) to conventional CT for sacroiliac joint bony morphology assessment in children.

**Methods:**

A prospective study was performed. Children who had (PET-)CT-scan underwent additional MRI. sCT-CT image quality was analyzed by two readers subjectively overall, semi-quantitatively in terms of cortical delineation, joint facet defects, growth plate fusion, ossified nuclei, lumbosacral transitional anomaly, and bony bridges, and quantitatively for disc space height, spinal canal width, and sacral vertebrae width and height. Cohen’s kappa and equivalence analyses with Bland–Altman plots were calculated for categorical and continuous measures respectively.

**Results:**

Ten patients were included (6 boys; aged 9–16 years; mean age 14 years). Overall sCT image quality was rated good. Semi-quantitative assessment of cortical delineation of sacroiliac joints, bony bridges, and joint facet defects on the right iliac and sacral sides showed perfect agreement. Correlation was good to excellent (kappa 0.615–1) for the presence of lumbosacral transitional anomaly, fusion of sacral growth plates, joint facet defect, and presence of ossified nuclei. sCT-CT measurements were statistically equivalent and within the equivalence margins (–1–1 mm) for intervertebral disc space height and spinal canal width.

Intra- and inter-reader reliability was excellent for quantitative assessment (0.806 < ICC < 0.998). For categorical scoring, kappa ranged from substantial to excellent (0.615–1).

**Conclusion:**

sCT appears to be visually equivalent to CT for the assessment of pediatric sacroiliac joints. sCT may aid in visualizing sacroiliac joints compared to conventional MRI, with the benefit that no ionizing radiation is used, especially important in children.

**Critical relevance statement:**

MRI-based synthetic CT, a new technique that generates CT-like images without ionizing radiation, appears to be visually equivalent to CT for assessment of normal pediatric sacroiliac joints and can potentially assess structural damage as it clearly depicts bony cortex.

**Key points:**

• MRI-based sCT is a new image technique that can generate CT-like images.

• We found that sCT performs similarly to CT in displaying bony structures of pediatric sacroiliac joints.

• sCT has already been clinically validated in the sacroiliac joints in adults.

• sCT can potentially assess structural damage from erosions or ankylosis as it clearly depicts bony cortex.

**Graphical Abstract:**

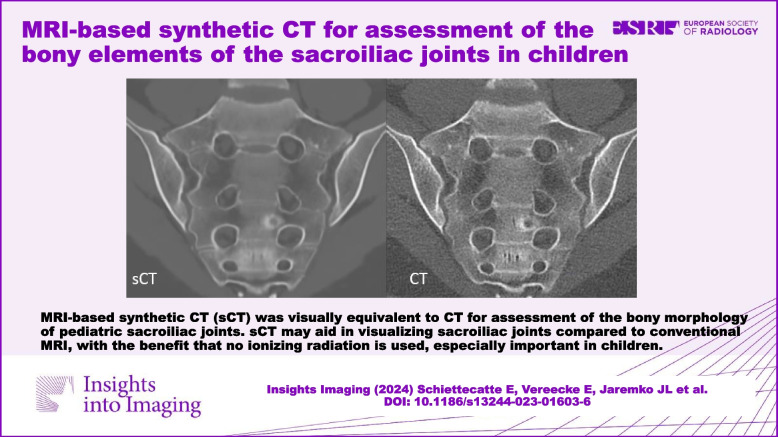

**Supplementary Information:**

The online version contains supplementary material available at 10.1186/s13244-023-01603-6.

## Introduction

Imaging of the sacroiliac (SI) joints in children is increasingly used for diagnosis and classification of juvenile spondyloarthritis (JSpA) [1]. Early diagnosis of sacroiliitis in children with JspA is important for therapy, especially with new biologic treatment options now available to delay progression and treat sacroiliitis in JspA [2, 3]. Historically the diagnosis of sacroiliitis was based on radiography. Radiography is not sensitive in early-stage disease [4] and has been replaced by MRI as the first line of investigation. MRI is excellent to detect active lesions of sacroiliitis and can also assess structural damage [2]. T1-weighted MRI images are the standard in evaluating structural lesions of sacroiliitis [5]. However, structural lesions including erosions, can be difficult to reliably assess on routine T1-weighted MRI scans in children [6]. The SI joint in adults is delineated as a sharply defined low signal black line on T1-weighted MRI images, which is rarely the case in children. Normal growth-related variations such as the absence of this black line representing the subchondral bone plate, blurring, and irregularity can mimic erosions, making this diagnosis more difficult in children [6, 7]. New ways to visualize and assess the bony structures of the SI joints on MRI would be helpful.

In most parts of the body, computed tomography (CT) is the method of choice to demonstrate bony anatomy [8]. In adults, CT has proven to be superior to MRI for assessing bone sclerosis and ankylosis in sacroiliitis [8]. Unfortunately, single-energy CT is not able to detect active lesions of sacroiliitis, which are key to diagnosing sacroiliitis according to the Assessment of SpondyloArthritis International Society (ASAS) criteria [9, 10]. CT can be used as a standard in adults in the evaluation of chronic sacroiliitis [1, 5, 11–13], but it cannot routinely be used in children due to radiation exposure [2, 8].

Synthetic CT (sCT), a new MRI technique, is a deep learning-based technology performing a 3-dimensional MRI-to-CT mapping, which is learned from paired MRI and CT data, generating ‘CT-like’ images without ionizing radiation. The sCT is generated from an axial 3D T1-weighted radiofrequency spoiled multiple gradient-echo sequence (T1MGE) [14]. This technology has previously been clinically validated for the SI joints in adults but has not yet been validated for use in children [9, 14, 15].

The purpose of this study is to assess the equivalence of MRI-based sCT to conventional CT in normal SI joints in children in a semi-quantitative and quantitative assessment of bony morphology.

## Materials and methods

This prospective study was approved by the local ethics committee and written informed consent was obtained from all patients and their parents. Authors without conflicts of interest had full control of the inclusion of any data and information submitted for publication. None of the data from study participants has been reported previously.

### Study patients

From May 2021 until June 2022, patients aged 6 to 18 years who underwent clinical CT of the pelvis or abdomen or whole-body PET-CT for any reason except for lower back pain, were asked to participate in our study. Patients admitted to the intensive care unit, very ill patients, patients who needed sedation for MRI, and immunocompromised patients were excluded. An additional MRI scan was done within approximately 1 month after the CT or PET-CT scan.

### CT protocol

CT and PET-CT images were acquired during clinical practice. CT scans were performed on Somatom Definition Edge Siemens Healthineers and Somatom Definition FLASH Siemens Healthineers. PET-CT was performed on Siemens Biograph mCT flow 20 PET/CT scanner (Siemens Healthcare, Erlangen, Germany) and GE Discovery MI 3ring scanner (GE Healthcare, Waukesha, WI, USA).

### MRI protocol

All MRI scans were performed on a 3.0-Tesla MRI unit (Prisma, Siemens Healthineers, Erlangen, Germany). An axial 3-dimensional T1-weighted radio-frequency-spoiled multiple gradient echo (3DT1MGE) sequence was performed: 2 echoes: repetition time/echo time 1/echo time 2: 7/2/3.5 ms, field of view 360 × 360 mm, acquisition matrix: 352 × 352, voxel size: 0.5 × 0.5 × 0.8 mm, acquisition time: 5 min 12 s.

### Synthetic CT reconstruction

sCT images were reconstructed with a commercially available software (BoneMRI Pelvic Region, version 1.4, MRIguidance BV). The software runs on-site and is connected to the hospital picture and archiving and communication system (PACS). The PACS automatically forwards the source MRI images to the sCT software, which reconstructs sCT images with a processing time of around 30 min. No manual input is required. The software reconstructed sCT images from two 3DT1MGE images derived from two different echoes using a deep learning method based on the U-net architecture. This method exploits local spatial contextual information in the multi-echo data to reconstruct the latent bone structures, which was learned using paired MRI and CT data. The resulting sCT image expresses radiodensity contrast in Hounsfield units (HU) values [14].

### Analysis/image assessment/definitions

CT and sCT were reconstructed in a paracoronal plane (parallel to the dorsal cortex of the S2 vertebral body) a true axial and a true sagittal plane, all with a slice thickness of 1 mm. Two radiologists (N.H. and E.S.) with 18 and 10 years of experience respectively, independently reviewed sCT and CT images. For the measurements, sCT and CT images were mixed and displayed in random order. Readers were blinded to clinical and demographic findings. Definitions were first defined in consensus on 5 other sCT and 5 other CT scans of pediatric patients, not included in the study because they did not have paired data.

Overall image quality, presence of lumbosacral transitional anomaly, and fusion of sacral vertebral growth plates at levels S1/S2 and S2/S3 were scored. Cortical delineation of the joint space at both iliac and sacral sides, presence of joint facet defects, ossified nuclei, and bony bridges were scored (Table 1). Quantitative analysis was performed by measuring the maximal diagonal width and height of the S1 and S2 vertebral bodies and the maximum height of the disc space at levels L5–S1, both on a midsagittal plane. The maximum width of the spinal canal at levels L5–S1 was measured in the axial plane (Table 2). Prior to quantitative analysis, the two readers reached a consensus on measurement methodology.

**Table 1:** Overview of planes, methods, level/side, and scoring

All planes	*Overall image quality:*		1 = Poor, impossible for visualization of bony structures	N/A
			2 = Impaired, difficult for visualization of bony structures	N/A
			3 = Good, for visualization of bony structures	
			4 = Excellent	
Paracoronal	*Lumbosacral transitional anomaly:*		0 = Not present	
			1 = Present	
Paracoronal	*Fusion of the growth plates of the sacrum:*	Level S1/S2	0 = Open	
			1 = Partially closed	
			2 = Completely closed	
		Level S2/S3	0 = Open	
			1 = Partially closed	
			2 = Completely closed	
Paracoronal	*Cortical delineation of the joint space:*	Iliac side (R/L)	0 = Not clearly visible, the iliac joint is not completely seen as a hyperdense cortical line	
			1 = Clearly visible, the iliac joint is clearly seen as a hyperdense cortical line	
		Sacral side (R/L)	0 = Not clearly visible, the sacral joint is not completely seen as a hyperdense cortical line	
			1 = Clearly visible, the sacral joint is clearly seen as a hyperdense cortical line	
Paracoronal	*Joint facet defect (*≥ *3 mm):*	Iliac side (R/L)	0 = Not present, no surface defect greater than or equal than 3 mm are present	
			1 = Present, there is one or more surface defect(s) greater or equal than 3 mm (the measurement of the defect can be parallel or perpendicular to the joint space)	
		Sacral side (R/L)	0 = Not present, no surface defect greater than or equal than 3 mm are present	
			1 = Present, there is one or more surface defects greater or equal than 3 mm (the measurement of the defect can be parallel or perpendicular to the joint space)	
Paracoronal	*Presence of ossified nuclei of the SI joint:*	R/L	0 = Not present	
			1 = Present and not fused	
			2 = Partially fused ossified nuclei	
Paracoronal	*Bony bridges of the SI joint:*	R/L	0 = Not present	
			1 = Present	

*Abbreviations*: *N/A* not applicable, *S* sacral, *L* lumbar, *R/L* right/left, *SI joint* sacroiliac joint

**Table 2:** Overview of quantitative analysis

Mid sagittal	*Maximum diagonal width* *of vertebral body from anterior to posterior*	S1	
Mid sagittal	*Maximum diagonal height of vertebral body from superior to inferior*	S1	
Mid sagittal	*Maximum diagonal width of vertebral body from anterior to posterior*	S2	
Mid sagittal	*Maximum diagonal height of vertebral body from superior to inferior*	S2	
Mid sagittal	*Maximum height of the intervertebral disc space*	L5-S1	
Axial	*Maximum spinal canal width*	L5-S1	

*Abbreviations*: *S* sacral, *L* lumbar

In order to investigate the intra-reader reliability, 1 reader (E.S.) repeated the measurements 1 month later (a delay added to avoid recall bias).

### Statistical analysis

An agreement between sCT and CT for the categorical measures was determined by Cohen’s kappa. Confusion matrices were generated for CT as the gold standard. For the continuous measures, differences between methods were analyzed with Bland–Altman plots and equivalence analyses. An equivalency design was chosen to prove whether the outcomes did not differ by more than a clinically or scientifically meaningful threshold [16]. For the equivalence analyses, the equivalence margin was set to 1 mm, which represents 1 voxel of a typical high-resolution CT scan.

### Inter- and intra-reader reliability

For the categorical measures, Cohen’s kappa was calculated, and confusion matrices were constructed. For the continuous measures, inter- and intra-rater reliability measures were obtained by calculating the intra-class correlation coefficient (ICC), using a two-way and one-way random model, respectively, for absolute agreement and a single measure.

All statistical analyses were performed using R 4.2.2.

## Results

In all patients, MRI was obtained within a maximum period of just over one month (range 5–33 days) after performing clinical CT or PET-CT scan. Six boys and 4 girls were included aged between 9 and 16 years (mean age 14 years), resulting in a total of 20 SI joints. There was no reported SI joint pathology in these patients.

**Fig. 1:**
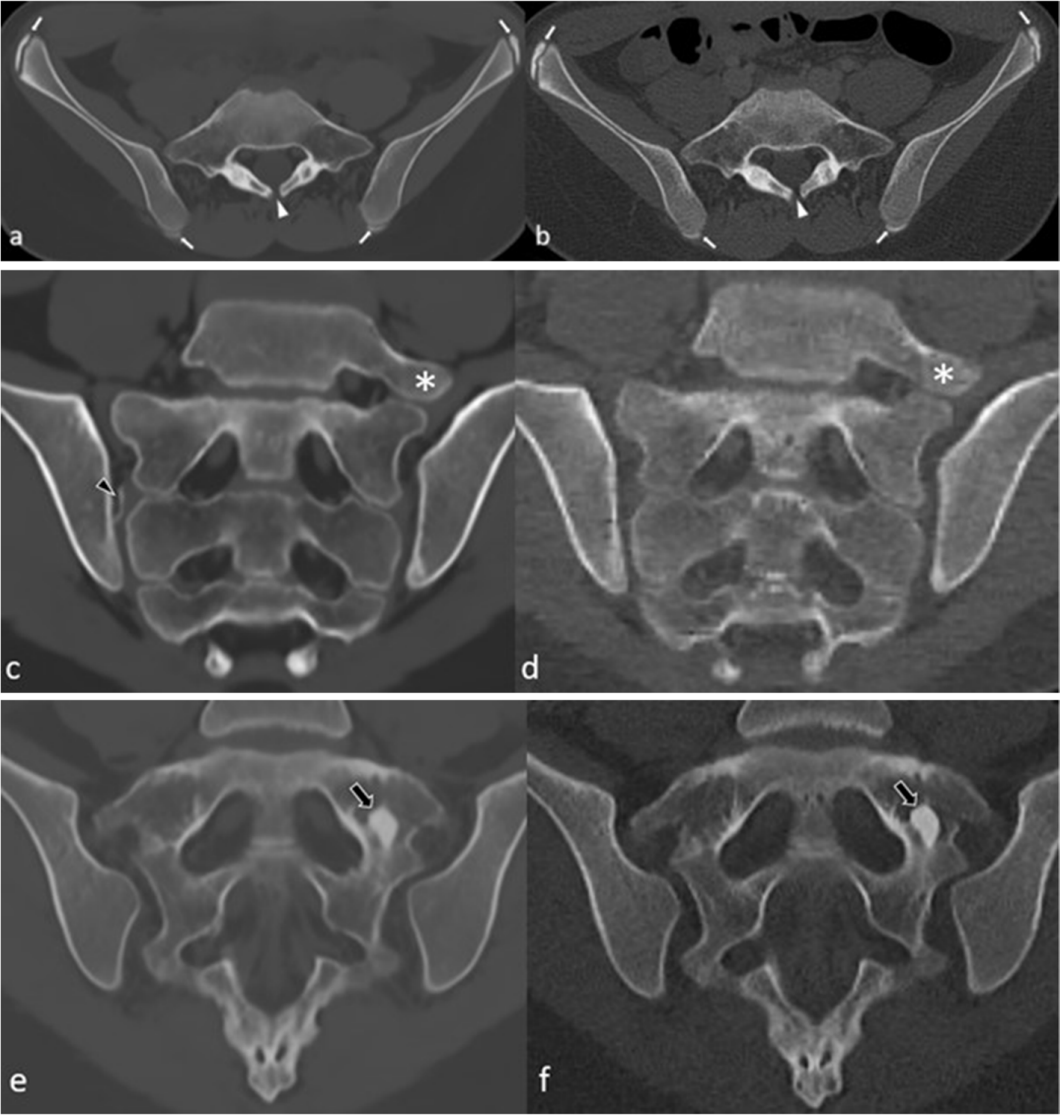
Some incidental findings were seen on both MRI-based synthetic CT (sCT) (left images **a**, **c**, and **e**) and CT (right images **b**, **d**, and **f**). **a**–**b** Spina bifida occulta of S1 (arrowhead) in a 15-year-old girl in an axial plane. Also note the ossification centers of the apophysis of the iliac crest that can be seen as well (arrows). **c**–**d** Lumbosacral transitional anomaly without bony fusion on the left side (asterisk) in a 12-year-old boy in a paracoronal plane. Also note the white line in the right SI joint (black arrowhead), consistent with an artifact on sCT (this was not seen on CT). **e**–**f** Enostoma in a 16-year-old female on the left side of S1 in a paracoronal plane (black arrows)

In addition to the features that were scored semi-quantitatively and quantitatively, some obvious other incidental findings were depicted on CT as well as on sCT, such as spina bifida occulta (2 cases), ossification centers of the apophysis of the iliac crest (4 cases) and an enostoma (1 case) (Fig. 1).

**Fig. 2:**
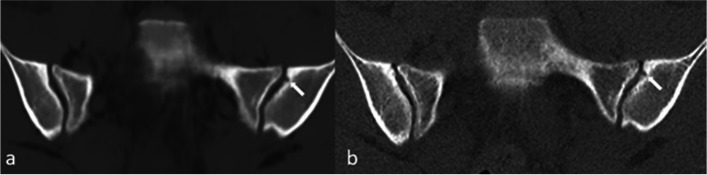
Joint facet defect ≥ 3 mm (arrows) in the left iliac joint in a 16-year-old girl in a paracoronal plane. **a** MRI-based synthetic CT (sCT). **b** CT

**Fig. 3:**
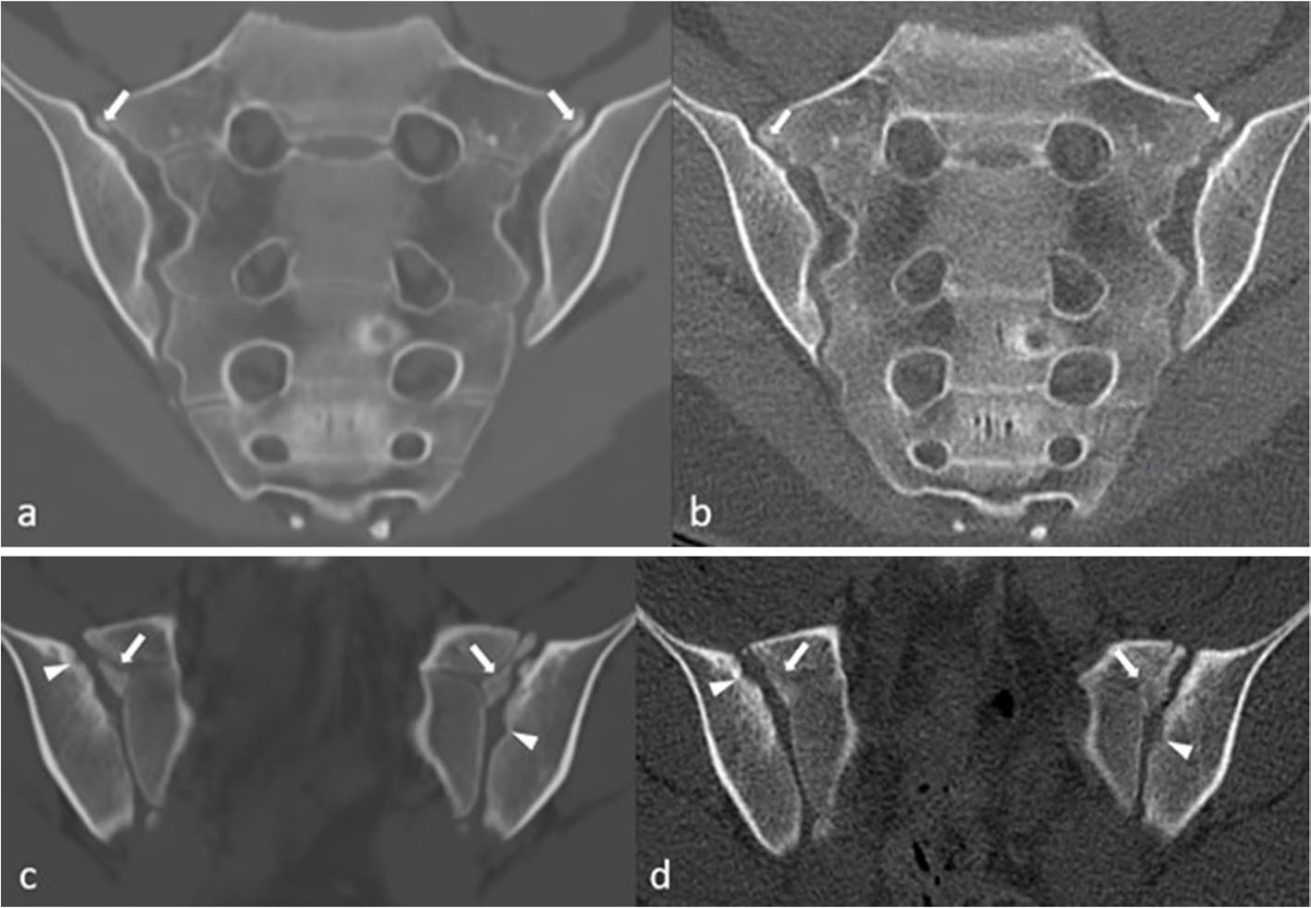
Comparison between MRI-based synthetic CT (sCT) (**a**, **c**) and CT (**b**, **d**) in a 15-year-old girl in a paracoronal plane. Arrows indicate ossified nuclei. Also note the irregularities seen on sCT and CT (arrowheads) on the iliac side of the sacroiliac (SI) joints, these did not meet the criterion of ≥ 3 mm for joint facet defect

One patient had a joint facet defect meeting the criterion of ≥ 3 mm, seen on both sCT and CT (Fig. 2). There were multiple irregularities seen on sCT and CT on the iliac and sacral side of the SI joint that did not meet the criterion of ≥ 3 mm (Fig. 3).

### Semi-quantitative assessment

Excellent image quality was scored on all sCT and CT images except for 1 sCT that was scored as good due to motion artifacts.

Agreement on the presence of features in categorical scoring was high. Kappa ranged from substantial (0.672) to excellent (1) (median = 0.672) [17], except for bony bridges on the left side, (kappa = 0. Table 3). However, this is due to low variability and small sample size, as the methods agreed on 9 out of 10 times for this measure (Table S1) [17]. Because of a lack of variability, we were unable to calculate a kappa for 8 measures. For these measures, there was a 100% agreement between sCT and CT.

**Table 3:** Cohen’s kappa (%) for the categorical scoring between synthetic CT (sCT) and CT

Measurement	Kappa (%)		
		Right side	Left side
*Lumbosacral transitional anomaly:*	100		
*Fusion of the growth plates of the sacrum S1/S2:*	83.9		
*Fusion of the growth plates of the sacrum S2/S3:*	84.8		
*Cortical delineation of the joint space of the iliac side:*		100^a^	100^a^
*Cortical delineation of the joint space of the sacral side:*		100^a^	100^a^
*Joint facet defect (*≥ *3 mm) on the iliac side:*		100^a^	61.5
*Joint facet defect (*≥ *3 mm) on the sacral:*		100^a^	100^a^
*Presence of ossified nuclei of the SI joint:*		67.2	67.2
*Bony bridges of the SI joint:*		100^a^	0

*Abbreviations*: *sCT* synthetic CT, *S* sacral; *SI joint* sacroiliac joint

^a^100% perfect agreement between sCT and CT, kappa could not be calculated because of lack of variability

### Quantitative assessment

**Fig. 4:**
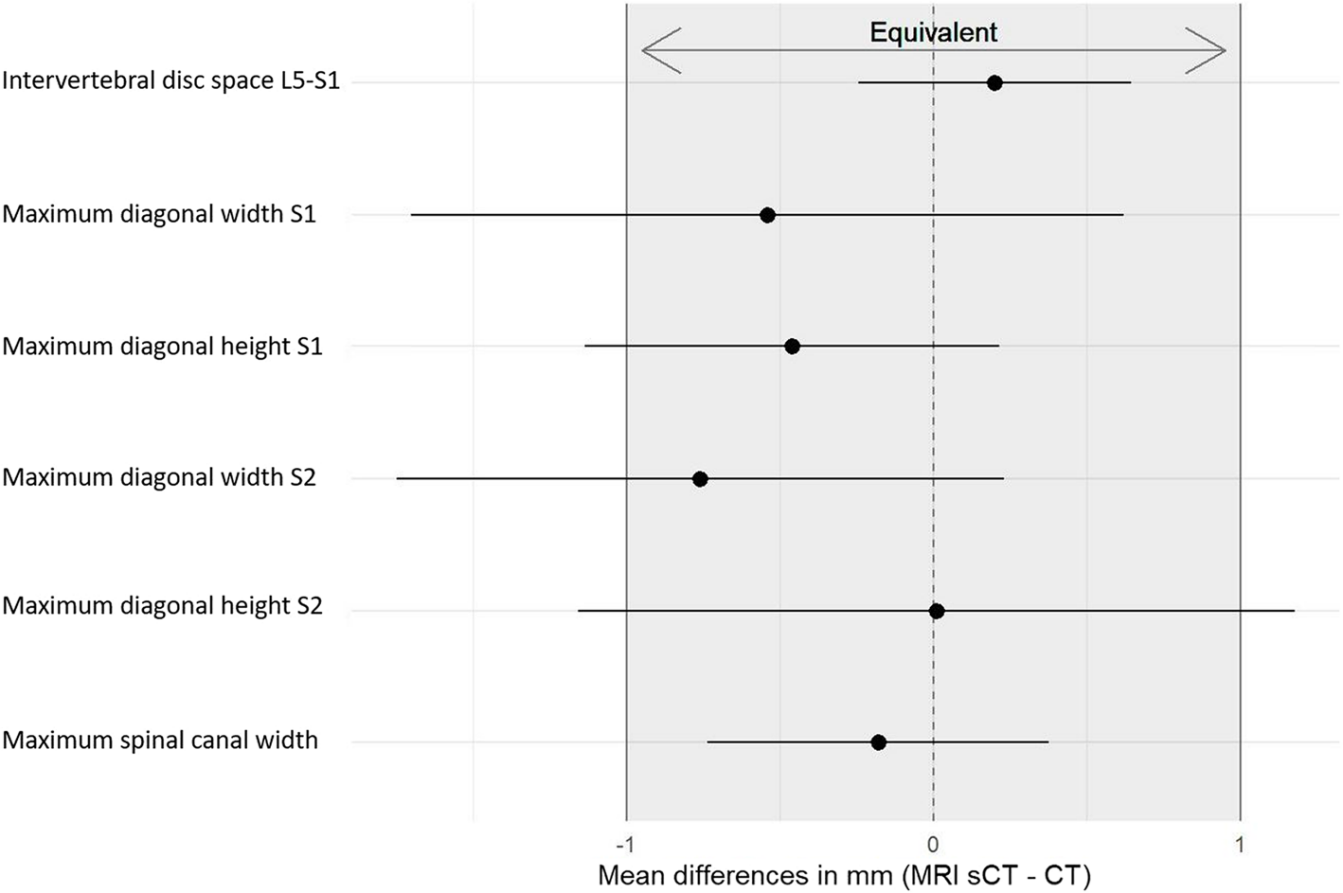
Equivalency plot of synthetic CT (sCT) to CT depicting the outcome difference in the geometrical measurements on sCT and CT. The plot indicates the equivalency of sCT to CT when the entire confidential interval (CI) is within the two margins (− 1 and 1 corresponding with an equivalency margin of − 1 mm and 1 mm, respectively). Shaded area, equivalency range; L, lumbar; S, sacral

In the Bland–Altman plots the differences between sCT and CT were plotted against the mean score for each measurement, showing that the deviations were independent of the size of the measurements (Fig. S1). For the 6 continuous measures, we could not find statistically significant differences in means in this sample. sCT was equivalent to CT for the geometric measurement of intervertebral disc space height and maximum spinal canal width. The measurements of S1 and S2 vertebral bodies were not within the equivalency margin of 1 mm. The equivalency plot of sCT to CT is shown in Fig. 4, indicating equivalency when the entire confidential interval (CI) is within the two margins (–1–1 mm). For the 6 continuous measures, equivalence was calculated (in mm): for diagonal width S1 (− 1.7–0.62), diagonal height S1 (− 1.13–0.21), diagonal width S2 (− 1.75–0.23), diagonal height S2 (− 1.16–1.18), intervertebral disc space L5–S1 (− 0.25–0.65), and spinal canal width (− 0.74–0.38).

### Inter- and intra-reader reliability

#### Semi-quantitative assessment

Inter- and intra-reader agreement on categorical scoring was generally high. Kappa ranged from substantial (0.615) to excellent (1), except for inter-reader reliability for bony bridges of the right SI joint and cortical delineation of the joint space of the left sacral side on sCT (both 0, again due to low variability and small sample size, as the readers agreed in 9 out of 10; Table 4). One reader scored cortical delineation as insufficient one-time due to motion artifacts on sCT. Because of a lack of variability, we were unable to calculate a kappa for inter-reader reliability in 15 measures and for intra-reader reliability in 16 measures (Table 4). For all these measures there was a 100% agreement between the readers.

**Table 4:** Cohen’s kappa (%) for the categorical scoring for inter- and intra-reader reliability of synthetic CT (sCT) and CT

Measurement	For inter-reader reliability						For intra-reader reliability					
	Kappa sCT (%)			Kappa CT (%)			Kappa sCT (%)			Kappa CT (%)		
		Right side	Left side		Right side	Left side		Right side	Left side		Right side	Left side
Lumbosacral transitional anomaly:	100			100			100			100		
Fusion of the growth plates of the sacrum S1/S2:	100			84.8			100			83.9		
Fusion of the growth plates of the sacrum S2/S3:	84.8			70.6			100			70.6		
Cortical delineation of the joint space of the iliac side:		100^a^	100^a^		100^a^	100^a^		100^a^	100^a^		100^a^	100^a^
Cortical delineation of the joint space of the sacral side:		100^a^	0		100^a^	100^a^		100^a^	100^a^		100^a^	100^a^
Joint facet defect (≥ 3 mm) on the iliac side:		100^a^	61.5		100^a^	100		100^a^	61.5		100^a^	100
Joint facet defect (≥ 3 mm) on the sacral:		100^a^	100^a^		100^a^	100^a^		100^a^	100^a^		100^a^	100
Presence of ossified nuclei of the SI joint:		83.1	83.1		67.2	100		100	100		65.5	100
Bony bridges of the SI joint:		0	61.5		100^a^	100^a^		100^a^	61.5		100^a^	100^a^

*Abbreviations*: *sCT* synthetic CT, *S* sacral, *SI joint* sacroiliac joint

^a^100% perfect agreement between the readers, kappa could not be calculated because of lack of variability

#### Quantitative assessment

ICCs were excellent for inter- and intra-reader reliability for all quantitative measurements [18]. The overall inter-reader ICC was 0.945 to 0.996 on sCT and 0.909 to 0.994 on CT. The intra-reader ICC was 0.806 to 0.998 on sCT and 0.946 to 0.955 on CT. In Table 5, the ICCs obtained for the combined measurements for the intra- and inter-reader reliability are displayed.

**Table 5:** Intraclass correlation coefficient (ICC) obtained for all the measurements for the inter- and intra-reader reliability of synthetic CT (sCT) and CT

	Inter-reader reliability		Intra-reader reliability	
Measurement	sCT	CT	sCT	CT
*Maximum diagonal width* *of vertebral body S1*	0.994	0.982	0.995	0.993
*Maximum diagonal height of vertebral body S1*	0.991	0.962	0.995	0.975
*Maximum diagonal width* *of vertebral body S2*	0.979	0.914	0.996	0.977
*Maximum diagonal height of vertebral body S2*	0.955	0.909	0.982	0.984
*Maximum height of the intervertebral disc space*	0.945	0.966	0.806	0.946
*Maximum spinal canal width*	0.996	0.994	0.998	0.995

*Abbreviations*: *ICC* intraclass correlation coefficient, *sCT* synthetic CT, *S* sacral

## Discussion

This prospective study was performed to test the equivalency of MRI-based sCT images with conventional CT images of (normal) SI joints in children. The sCT images were generated from the 3DT1MGE sequence using a deep learning-based method aiming at specific visualization of the osseous morphology by HU estimation [14].

We found that overall image quality was good for all bony structures on sCT and CT. Osseous morphology was correctly visualized on sCT for detection and visualization of lumbosacral transitional anomalies, fusion of sacral growth plates, cortical delineation of the SI joint, presence of ossified nuclei of the SI joint, and joint facet defects of the SI joint. Cortical delineation of the joint space was equally well seen on sCT and CT.

**Fig. 5:**
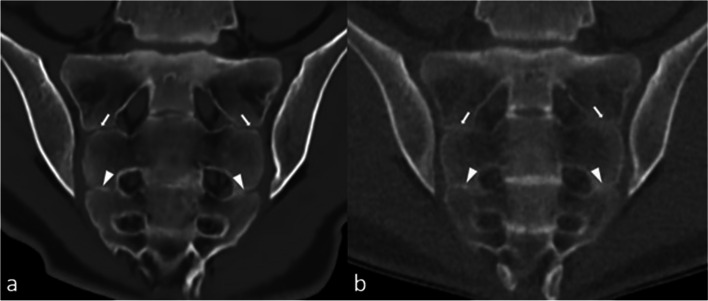
Comparison between MRI-based synthetic CT (sCT) (**a**) and CT (**b**) in a 13-year-old boy in a paracoronal plane. Arrows indicate open growth plates at the levels S1–S2 and arrowheads indicate partially closed growth plates at the S2–S3 level

Subjectively, in some cases, the sCT images even demonstrated sharper cortical delineation than the CT and PET-CT images (Fig. S2). This made ossified nuclei and growth plates better visualized and more sharply delineated on sCT than on CT (Figs. 3, 4, and 5), explaining partly the variability in measurement of S1 and S2 vertebral bodies between sCT and CT. Suboptimal CT scan images may be due to the use of low-dose protocols conforming to the ALARA principle (as low as reasonably achievable), in which the lowest possible radiation dose to achieve the clinical diagnosis is used, which does not necessarily provide excellent images for evaluation of bony structures [19, 20]. This may explain why measurements of S1 and S2 vertebral bodies were not within the 1 mm equivalency margin, especially the endplate of the S2 vertebral body which was sometimes difficult to see on a midsagittal plane.

In this study, we set a very strict 1-mm equivalence margin; all measures would have been within an equivalence margin of 2 mm.

The ongoing ossification process in children can result in unsharp cortical margins, making cortical assessment difficult. Multiple irregularities of the SI joints were seen in our study that did not meet the prespecified size definition of ≥ 3 mm (Fig. 3). Only 1 cortical irregularity met the definition and was observed on sCT as well as on CT by both readers (Fig. 2).

This is the first study using MRI-based sCT in children. In adults, several studies were published comparing sCT and CT in the SI joints, spine, and pelvis [9, 15, 21–26]. In a study of the lumbar spine and hips, geometric analysis of MRI-based sCT showed similar measurements in comparison to CT [15, 25]. Jans et al. showed that the diagnostic accuracy of sCT was higher than that of T1-weighted MRI sequences for detecting erosions, sclerosis, and ankylosis in adult patients suspected of having sacroiliitis [9]. Using sCT in children can be of great benefit in the assessment of sacroiliitis as assessing pediatric SI joints is very challenging on MRI, mainly due to normal variability. In children, blurring and irregularity of the SI joint line is frequently seen on classical T1-weighted sequences, making it difficult to detect erosions. sCT could definitely improve the assessment of joint delineation and bone erosions in pediatric SI joints [6]. Cortical delineation of the SI joint was excellent in all but one patient (who had motion artifacts) in our study on both sCT and CT.

sCT has a key advantage in that it can be scanned in one examination together with the classical MRI sequences, allowing assessment of active and structural lesions concomitantly and thus possibly better diagnostic interpretation of sacroiliitis in children with JSpA. Scan time is just 5 min 12 s longer when including the sCT sequence. Importantly, sCT comes with no ionizing radiation, which is essential in the younger population as the radiosensitive reproductive organs are in close proximity to the SI joints. Even though low-dose CT shows promising results in adults with SpA, in pediatric patients it is particularly preferable to perform an examination without ionizing radiation such as sCT [11–13].

sCT is also a 3D modality, thus allowing for images to be reconstructed in all planes, which could be especially beneficial to evaluate the rather complex SI joints [21].

Ankylosis and bony bridges of the SI joint are known structural lesions in sacroiliitis [3, 4, 10], however they were not expected in this normal population. In our study, bony bridges were scored in only 1 patient on sCT on the left side, not on CT, resulting in a kappa value of 0 (Table 3) (Table S1). Bony bridge-like features are a known artifact on sCT and a potential pitfall that has already been described in adults when using sCT for the diagnosis of sacroiliitis. Morbée et al. have shown that the vacuum phenomenon can be falsely seen as the bony bridge on sCT in SI joints [21]. In our case too, this rather thin white “artifactual” line crossing the SI joint on sCT (Fig. 1b.) did not mimic true ankylosis as can be seen in sacroiliitis, also no other features of sacroiliitis were present. In our normal study group, we also detected an incidental enostoma (Fig. 1e–f.) near the SI joint, which was equally seen on CT and sCT. This is promising as sCT might be used as well for assessing sclerosis as a structural lesion of sacroiliitis [21].

Our study has some limitations. The main limitation is that we did not image children with sacroiliitis, hence our study is limited to assessing image quality for normal appearances and normal variation. CT scans are not commonly performed in children with sacroiliitis due to radiation dose. Also, CT and MRI did not take place at the same day. The additional MRI scan was obtained within approximately 1 month after the CT scan. Also, different kinds of CT, including PET-CT were used, depending on the clinical question.

Another limitation is the low number of examined patients in this study. CT is in most children replaced by ultrasound or MRI in abdominal and pelvic pathology. Moreover, if an abdominal or pelvic CT was nevertheless performed, critical illness prevented these children from undergoing an additional MRI examination within one month. In this study, the youngest patient was 9 years old. In even younger age groups sedation might even be necessary, which was not approved by the ethics committee.

Future studies in larger groups of children, also including pathological scans of the SI joint, are required to further examine the added value of sCT for assessment of structural lesions (erosions, ankylosis, and sclerosis) of sacroiliitis on sCT in pediatric SI joints in comparison with the classically used MRI sequences and CT as gold standard where available.

## Conclusion

By all our measures in a group of normal children, sCT was visually equivalent to CT for assessment of the bony morphology of pediatric sacroiliac joints. In a clinical setting, adding sCT to the MRI protocol may have additional value as it allows for easily visually interpretable visualization of the bony structures of the sacroiliac joints in children without the use of ionizing radiation.

### Supplementary Information

**Additional file 1: Table S1.** Contingency table for categorical scoring of bony bridges of the SI joint (left side) on sCT and CT. **Fig. S1.** Bland–Altman plots for maximum diagonal width (left) and height (right) of S1; maximum diagonal width (left) and height (right) of S2; maximum height of the intervertebral disc space L5–S1 and maximum spinal canal width. **Fig. S2.** Comparison of MRI-based synthetic CT (sCT) (left images a.- c.- e.) and CT (right images b.- d.- f.) in a 15-year-old girl (a.- b.), a 12-year-old boy (c.- d.) and a 15-year-old boy (e.- f.) in a paracoronal plane.

## Data Availability

The datasets used and analyzed during the current study are available from the corresponding author on reasonable request.

## Abbreviations

3DT1MGE: 3-Dimensional T1-weighted radio-frequency-spoiled multiple gradient echo
AI: Artificial intelligence
ALARA: As low as reasonable achievable
ASAS: Assessment of SpondyloArthritis International Society
CT: Computer tomography
HU: Hounsfield units
ICC: Intra-class correlation coefficient
JSpA: Juvenile spondyloarthritis
MRI: Magnetic resonance imaging
PACS: Picture and archiving and communication system
PET: Positron emission tomography
sCT: Synthetic CT
SI: Sacroiliac
STIR: Short tau inversion recovery
