# Supplementary material for: MRI-based synthetic CT for assessment of the bony elements of the sacroiliac joints in children
Source: Insights Imaging. 2024 Feb 18;15:53. doi: 10.1186/s13244-023-01603-6 (PMC10874918; doi:10.1186/s13244-023-01603-6)

|  |  | CT | |  |
| --- | --- | --- | --- | --- |
|  |  | Bony bridges present on CT | Bony bridges not present on CT | Total |
| sCT | Bony bridges present on sCT | 0 | 1 | 1 |
|  | Bony bridges not present on sCT | 0 | 9 | 9 |
|  | Total | 0 | 10 | 20 |
| *Abbreviations: sCT (Synthetic CT); SI joint (sacroiliac joint)* | | | | |

**MRI-based synthetic CT for assessment of the bony elements of the sacroiliac joints in children**

**ELECTRONIC SUPPLEMENTARY MATERIAL**

**Table S1.** Contingency table for categorical scoring of bony bridges of the SI joint (left side) on sCT and CT.

**Fig. S1.** Bland-Altman plots for maximum diagonal width (left) and height (right) of S1; maximum diagonal width (left) and height (right) of S2; maximum height of the intervertebral disc space L5-S1 and maximum spinal canal width.


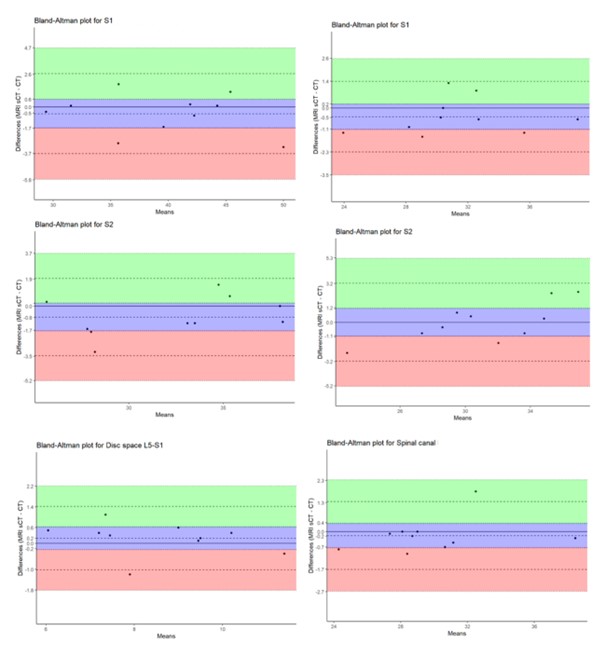


**Fig. S2.** Comparison of MRI-based synthetic CT (sCT) (left images a.- c.- e.) and CT (right images b.- d.- f.) in a 15-year-old girl (a.- b.), a 12-year-old boy (c.- d.) and a 15-year-old boy (e.- f.) in a paracoronal plane.


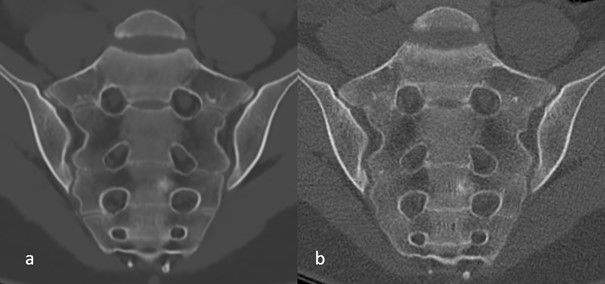


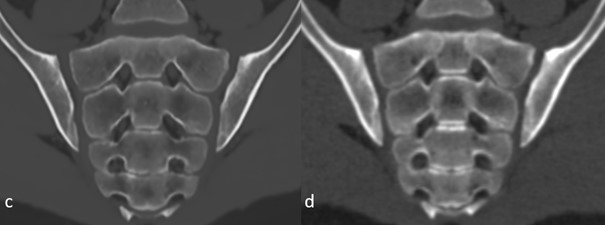


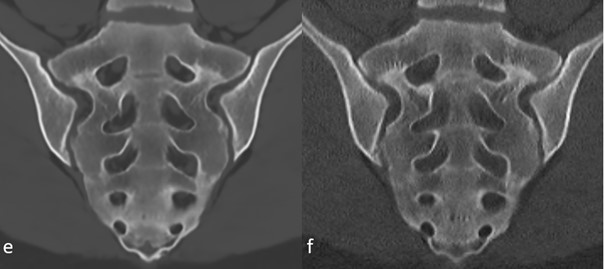

Supplement: Supplementary file 1 — Additional file 1: Table S1. Contingency table for categorical scoring of bony bridges of the SI joint (left side) on sCT and CT. Fig. S1. Bland–Altman plots for maximum diagonal width (left) and height (right) of S1; maximum diagonal width (left) and height (right) of S2; maximum height of the intervertebral disc space L5–S1 and maximum spinal canal width. Fig. S2. Comparison of MRI-based synthetic CT (sCT) (left images a.- c.- e.) and CT (right images b.- d.- f.) in a 15-year-old girl (a.- b.), a 12-year-old boy (c.- d.) and a 15-year-old boy (e.- f.) in a paracoronal plane. [file 13244_2023_1603_MOESM1_ESM.docx]
